# Annotations

**Published:** 1895-11-23

**Authors:** 


### Annotations.

### A Statue for Professor Huxley.

The Huxley Memorial Committee are divided among themselves as to whether a statue of the great Professor, or a scholarship bearing his name, will be the more appropriate monument. Surely there can be no two opinions. Whilst Huxley was alive, everybody who had not seen him wanted to see him, and everybody who had seen him once never forgot his face and figure again. Until one had seen Huxley one could never fully understand and realise the man. Once seen he was understood, and understood as a friend for evermore. If not merely as a scientific man, then as a typical John Bull Englishman, Huxley's figure should be perpetuated in immemorial stone. His erect form, his firmly clenched hands when he was roused, his strong mouth and chin, his truculent nose, his fine bold forehead, his fearless look and mien, everything conspired in him to show the breadth and the narrowness, the weakness and the irresistible strength of which the typical Englishman is the representative in the modern world. A scholarship by all means let us have if there be money enough after the statue is paid for; but a statue we must insist upon even if a fresh committee should have to be started for the purpose. Our warriors we have naturally commemorated in stone; our statesmen also, and even some of our preachers and philanthropists. Science, too, has her heroes, whose memories can only be adequately preserved for future generations by a permanent exhibition of their forms and features. Such a scientific hero was Huxley; or, if he was not, then science never had a hero, and never will have one. A statue, and a statue of heroic size, is the only memorial which will satisfy the imagination of the brave and admiring nation of which Huxley was so typical a representative.

### The New Broom at Walton-on-Naze.

Some time ago we had occasion to draw attention to the presence of diphtheria at Walton-on-Naze. Of course there were protests, and it appeared that the deaths took place from "infectious quinsey," but we need not hesitate to agree with the county medical officer for Essex in his advice that "the only safe plan is to regard them (cases of infectious quinsey) as cases of diphtheria, and insist upon all precautions being taken to prevent the disease spreading." Owing to the illness of the local medical officer of health, a substitute was appointed on September 26th, from whose report on the progress of affairs, dated November 1st (that is, a month after his appointment!) we gather that the health of the town is now "good; no deaths and no cases of diphtheria since August 7th. No typhoid, no small-pox, no scarlet fever, and no measles." This is, indeed, a demonstration of the efficacy of new brooms. But although there has been no diphtheria, and no deaths during the whole month from "infectious quinsey," there have been nearly a dozen cases during the month of this disease. Deaths from bad throats there may not have been, but in a place of only 1,600 inhabitants that is nothing. Calculated at the London rate the deaths in Walton-on-Naze from zymotic disease (small-pox, scarlet fever, measles, diphtheria, &c.), should only have amounted to about four in the whole year. The medical officer, then, has but small right to proclaim the health of the town as "good" because no deaths have taken place from this disease in any one month. As these cases are apparently being—very properly—dealt with as diphtheria, let us consider for a moment what is the meaning of twelve cases in a month in a population of 1,600. We think we are suffering greatly in London from diphtheria, and we do not hesitate to bemoan the fate of the little ones, but the notifications during October only amounted to one in every 2,730 persons living in London at the time, whereas the notifications of this Walton-on-Naze disease amounted—according to the report of the acting medical officer of health—to one in every 133 people in that little town. Whatever its fatality may be, the disease itself was far more prevalent last month than diphtheria was in London.

### Sleep and its Enemies.

The action of the Vestry of St. Mary Abbotts, Kensington, in trying to persuade the London County Council to take some steps to mitigate the intolerable noises of the streets, has led to much and very diverse comment in the press. Perhaps it is not to be wondered at that some papers should be sore tempted to make political capital out of the fact that most of those who make the vilest noises are members of the least wealthy classes, and that the quarter from which this protest comes is one in which wealth abounds; but it certainly is a matter for some surprise that a fairly level-headed journal like the *Spectator* should take this cue, preaching to the "fastidious classes" the "duty of self-renunciation in deference to popular taste," talking of the "tyranny" of "aristocratic senses," and suggesting the fearful things which might happen "if once the high-sniffing classes got the reins in their hands." All this is mere bunkum and fine writing, for those who know the London poor best know well that one of the great hardships which they have to bear is the constant unmitigated riot amid which so many of them have to live and sleep. To the rich noise, no doubt, is chiefly an æsthetic nuisance, and to the very rich it is capable of mitigation in case of illness; but to the middle classes and the poor, to all who are productive, and whose powers of production depend on quiet in their hours of rest, the more specially objectionable noises of the streets are a cause of suffering and loss. We cannot hold with the *Spectator* that "the great heart of the people is not fastidious," or that they find as "their greatest pleasure noise—miscellaneous, sociable, commonplace noise." The fact is that the people who make our streets so hideous, and so disturb the peace of the working population, are but a small minority, a perfectly insignificant minority, in comparison with the people they annoy. Medical men and nurses know well the added hardship suffered by the poor in illness in consequence of the ever-present noises to which they are exposed; and, so far from agreeing with the *Spectator* that this is a case of class distinction and attempted class oppression, we believe that none would profit more than the poor from some mitigation of the noises—we speak here chiefly of street cries—by which in health their repose is spoilt, and in illness their life is made a burden.

